# Topical Treatments in Reducing *Propionibacterium Acnes* Infection in Shoulder Surgery: Protocol for a Systematic Review and Meta-analysis

**DOI:** 10.29337/ijsp.174

**Published:** 2022-06-09

**Authors:** Yash Sewpaul, Brook Leung, Alexander W. Hartland, Sandeep Krishan Nayar, Mustafa S. Rashid

**Affiliations:** 1 Lancaster Medical School, Lancaster University, LA1 4YW, GB; 2 Royal London Hospital, Whitechapel Rd, London, E1 1FR, UK; 3 Northampton General Hospital, Cliftonville, Northampton, NN1 5BD, UK; 4 Nuffield Department of Orthopaedics, Rheumatology, and Musculoskeletal Sciences, Windmill Road, Oxford, OX3 7LD, UK

**Keywords:** Shoulder surgery, Infection, Topical preparations, Propionibacterium acnes, Culture

## Abstract

**Introduction:**

*Propionibacterium acnes* is a gram-positive anaerobe that is found on the dermis and epidermis of the shoulder and is the most commonly identifiable cause of periprosthetic shoulder joint infection. Various topical strategies have been investigated to reduce the prevalence of *P acnes*, with several demonstrating efficacy in reducing the positive culture. The aim of this systematic review and meta-analysis is to review the literature to assess the effectiveness of topical preparations in reducing the prevalence of *P acnes* in shoulder surgery.

**Methods:**

The study protocol was designed and registered prospectively on PROSPERO (International prospective register for systematic reviews). Databases used for the literature search will include MEDLINE, EMBASE, PsycINFO, and The Cochrane Library. Randomised controlled trials (RCTs) evaluating the use of any topical preparation against placebo, in all types of shoulder surgery, will be included. Our primary outcome is the number of colony forming units of P acnes. Secondary outcomes will include adverse events such as skin irritation, wound dehiscence, and the incidence of revision surgery due to infection. The Cochrane Risk of Bias Tool 2.0 and Jadad score will be used to assess the quality of methodology of the studies. Statistical analysis will be used to assess inconsistency and bias across included studies. Comparable outcome data will be pooled and analysed quantitatively or qualitatively as appropriate.

**Ethics and dissemination:**

No ethical clearances required for this study. This systematic review and meta-analysis will be published in a peer-reviewed journal.

**Highlights:**

- Various topical strategies have been investigated to reduce the prevalence of P acnes, the most common identifiable cause of periprosthetic shoulder joint infection, with several demonstrating efficacy in reducing the positive culture.
- This systematic review and meta-analysis evaluates the effectiveness of topical preparations in reducing the prevalence of P acnes in shoulder surgery.
- Our primary outcome is the number of colony forming units of P acnes.

**Registration:**

PROSPERO 2022 CRD42022310312.

## 1. Introduction

*Propionibacterium* (also *Cutibacterium*) acnes is an anaerobic, gram-positive bacterium that inhabits the sebaceous glands of the skin and sebum-rich hair follicles [1]. There is an increased prevalence of *P acnes* found on the shoulder and its surrounding regions due to the increased number of hair follicles and sebaceous glands, and there is therefore a higher prevalence of *P acnes* culture on the shoulder than other common orthopaedic surgical sites such as the knee or hip [1,2,3]. *P acnes* is found in both the epidermal and dermal layers of the skin and is one of the most common causative pathogens of postoperative infection in open shoulder surgery [4]. Data is however limited on the incidence of *P acnes* infection in shoulder arthroscopy. A significant proportion of patients undergoing shoulder surgery have a positive skin and/or joint culture for *P acnes*, and a small proportion of these patients go onto develop a postoperative *P acnes* infection [5]. P acnes is the most commonly identifiable cause of periprosthetic joint infection, with literature reporting positive culture rates between 20%–70% [6,8]. Other microorganisms found to be responsible for periprosthetic joint infections include; high-virulent pathogens in early infections –*Staphylococcus aureus, streptococci, and enterococci –* and low-virulent organisms in delayed infections *– coagulase-negative staphylococci* or *cutibacterium species* [9].

Various topical strategies have been studied to reduce the prevalence of *P acnes* on the shoulder girdle before surgery, such as benzoyl peroxide, topical antibiotics such as clindamycin phosphate, chlorhexidine gluconate and Betadine (Purdue Pharma LP, Stamford, CT, USA) [7,8,10,11,12,13]. Betadine and chlorhexidine gluconate have been shown to be ineffective at reducing the prevalence of *P acnes* at the surgical site [13]. This is most likely because these specific topical preparations cannot reach the sebaceous glands, and once a skin incision is made, *P acnes* can enter the surgical wound.

Benzoyl peroxide (BPO) is a bactericidal agent commonly used for treating acne vulgaris and has been shown in multiple randomised studies to reduce the prevalence of *P acnes* on the shoulder [10,12,14]. Application of 5% BPO three days prior to surgery has been shown to effectively reduce *P acnes* culture [11,12,14]. There is however an issue of patient compliance with this particular management as some patients can experience mild pruritus when using 5% BPO [14,15,16]. Moreover, the combination of 5% BPO with 1.2% clindamycin phosphate gel has also been shown to reduce *P acnes* colonisation [8]. Most recently, hydrogen peroxide, the active agent in BPO, has been shown to reduce *P acnes* in patients undergoing arthroscopic shoulder surgery, but dermal application after skin incision does not significantly reduce the prevalence of *P acnes* during shoulder arthroplasty [7,13].

The aim of this systematic review and meta-analysis is to evaluate the use and outcomes of topical treatments to reduce *P acnes* prevalence in shoulder surgery. Our primary outcome will be the number of colony forming units (CFU). Secondary outcomes will include adverse events related to both infection and application of topical preparations, such as skin irritation, wound dehiscence, and the incidence of revision surgery due to infection.

## 2. Methods

This study protocol was designed and registered prospectively on the PROSPERO (International prospective register for systematic reviews) database (Ref: CRD42022310312) [17]. The protocol is reported according to the Preferred Reporting Items for Systematic reviews and Meta-Analysis Protocol (PRISMA-P) [18,19].

### 2.1. Eligibility criteria

#### 2.1.1. Study design

Only randomised controlled trials will be included. All other trial designs will be excluded.

#### 2.1.2. Participants

We will include studies with humans of any age undergoing any type of surgery to the shoulder. This will include both arthroscopic and open techniques. Example open procedures may include shoulder arthroplasties and the Latarjet procedure. Example arthroscopic procedures may include diagnostic arthroscopies and rotator cuff repairs.

#### 2.1.3. Intervention and comparators

The intervention of interest is any topical treatment or preparation for the reduction of *P acnes*. We will include all individual methods of timing, dose, and area of application. The comparator can include any form of placebo or no treatment.

#### 2.1.4. Outcomes

The primary outcome of interest will be the number of colony forming units (CFU) of *P acnes*. Other measures of infection such as total viable counts (CFU/ml) may also be included.

Secondary outcomes examined will include adverse events related to infection and application of topical preparations, such as skin irritation, wound dehiscence, and the incidence of revision surgery due to infection.

#### 2.1.5. Timing

No restrictions regarding timing of the study.

#### 2.1.6. Setting

No restrictions regarding setting of the study.

#### 2.1.7. Language

Studies of all languages will be included. Any titles requiring translation into English will be included in the appendix.

### 2.2. Information sources

Our search strategy involved the following bibliographic databases; MEDLINE, EMBASE, PsycINFO and The Cochrane Library. The search strategy will be carried out and reported according to the Preferred Reporting Items for Systematic reviews and Meta-Analysis literature search extension (PRISMA-S) [20].

#### 2.2.1. Search strategy

No restrictions will be placed on publication date or language. Randomised controlled trial filters from the Cochrane group will be used to increase precision and sensitivity when searching. Utilised search terms are included in the appendix. References from published systematic reviews investigating the same or similar topic will also be manually searched for relevant included studies. On searching the PROSPERO database, no ongoing or recently completed systematic reviews on this topic were found.

### 2.3. Study records

#### 2.3.1. Data management

All literature search results will be combined and collected in Endnote X9 (Clarivate Analytics), and duplicate articles will be removed. The titles and abstracts from returned search results will be screened by two independent reviewers. This process will be conducted independently, and consensus will be sought prior to full text review. The final inclusion will then be determined by a full text review of articles that meet all eligibility criteria.

#### 2.3.2. Data collection process

Data extraction will involve two independent reviewers: one reviewer will extract data using a standardised proforma, and the second reviewer will check the extracted data for inaccuracies. Any discrepancies in data extraction will be resolved by discussion and the involvement of a third reviewer as needed. Should there be any unclear or missing data, or any required additional information needed, we will attempt to contact individual study authors. Data capture will be organised on Microsoft Excel and Review Manager (RevMan version 5.3) used as a software tool for data management.

#### 2.3.3. Data items

Data extraction will include study design, patient cohort, study characteristics, the topical preparation, dose and timing of application, control group intervention, primary outcome measures, and any secondary outcome measures. Mean and standard deviations will be extracted for all outcome measures. Data on adverse events will also be extracted.

### 2.4. Outcomes and prioritisation

#### 2.4.1. Primary outcome

The primary outcome of interest will be the number of CFUs of *P acnes*. Other outcome measures relevant to positive culture growth in vitro will also be included.

#### 2.4.2. Secondary outcomes

Secondary outcomes examined will include adverse events related to both infection and application of topical preparations, such as skin irritation, wound dehiscence, and the incidence of revision surgery due to infection.

### 2.5. Risk of bias of individual studies

To assess individual studies for potential bias, the Cochrane collaboration Risk of Bias tool will be used [21]. Five domains are used to categorise bias, with a level of risk is assigned to each domain (high risk, low risk, or some concerns). Within each domain, signalling questions will be posed to guide interpretation of bias. An overall risk of bias is then generated for each study. The Jadad scale will also be used as an additional method for assessing bias [22]. A maximum of five points can be given by the Jadad scale. A maximum of two points can be given for randomisation – a point can be given for a study being randomised and a further point if an appropriate method of randomisation is used. Two points can be given for blinding – a point if the study has stated the use of blinding and a further point if an appropriate method of blinding is used. If all patients involved in the trial have been accounted for, a final point is awarded.

### 2.6. Data synthesis

#### 2.6.1. Quantitative synthesis

A forest plot will be used to synthesise data related to culture of *P acnes* if the method in which they were recorded is comparable. Standardised mean differences and inverse variance statistical analysis will be used to summarise continuous variables, such as colony forming units. Heterogeneity is expected between studies; hence a random effects model will likely be used for analysis. The chi-square test and I^2^ statistic will be used to quantify heterogeneity. Any dichotomous data presented will be measured for effect using odds ratios. Review Manager (RevMan version 5.3) will be used as a software tool for data management and statistical analysis.

#### 2.6.2. Qualitative synthesis

Data will be reported descriptively if outcome measures are not comparable across studies, the heterogeneity is too high, or the incidence rate is too low for pooled statistical analysis.

#### 2.6.3. A priori subgroup analyses

There are no predetermined subgroup analyses. Subgroup analyses may be possible based on the topical treatment used, as well as, the method of timing, dose, and area of application.

#### 2.6.4. Meta-bias

A funnel plot will be used to assess publication bias of included studies investigating our primary outcome. Selective reporting will be assessed by reviewing any available trial registrations or trial protocols to compare the pre-defined intended outcomes with those analysed and reported in the final manuscript. The risk of bias for each study will be assessed as previously described. To measure inconsistency, a statistical analysis of heterogeneity will be used to assess bias across studies.

#### 2.6.5 Confidence in cumulative estimate

The Grading of Recommendations Assessment, Development and Evaluation (GRADE) approach will be used to assess the strength of the body of evidence [23,24,25]. Outcomes will be assessed as being of very low, low, moderate, or high certainty.

## Appendix

Search terms for MEDLINE

1. Randomised controlled trial.pt
2. Controlled clinical trial.pt
3. Randomised.ab,ti
4. Placebo.ab,ti
5. Randomly.ab,ti
6. Trial.ti
7. Clinical trial.mp
8. 1 OR 2 OR 3 OR 4 OR 5 OR 6 OR 7
9. Shoulder.ab,ti
10. Surgery.ab,ti
11. Procedure.ab,ti
12. Surgical.ab,ti
13. Repair.ab,ti
14. Reconstruction.ab,ti
15. Operative.ab,ti
16. Operation.ab,ti
17. Arthroplasty.ab,ti
18. Arthroscop*.ab,ti
19. Glenohumeral.ab,ti
20. Glenoid.ab,ti
21. Humerus.ab,ti
22. Humeral.ab,ti
23. Acromioclavicular.ab,ti
24. Clavicle.ab,ti
25. Acromion.ab,ti
26. Coracoid.ab,ti
27. Scapula.ab,ti
28. 9 OR 10 OR 11 OR 12 OR 13 OR 14 OR 15 OR 16 OR 17 OR 18 OR 19 OR 20 OR 21 OR 22 OR 23 OR 24 OR 25 OR 26 OR 27
29. Propionibacterium acnes.ab,ti
30. P acnes.ab,ti
31. Cutibacterium acnes.ab,ti
32. C acnes.ab,ti
33. 29 OR 30 OR 31 OR 32
34. Topical.ab,ti
35. Betadine.ab,ti
36. Povidone-iodine.ab,ti
37. Iodopovidone.ab,ti
38. Iodophor.ab,ti
39. Chlorhexidine.ab,ti
40. CHG.ab,ti
41. Isopropyl alcohol.ab,ti
42. ChloraPrep.ab,ti
43. DuraPrep.ab,ti
44. Benzoyl peroxide.ab,ti
45. BPO.ab,ti
46. Hydrogen peroxide.ab,ti
47. Sterile.ab,ti
48. 34 OR 35 OR 36 OR 37 OR 38 OR 39 OR 40 OR 41 OR 42 OR 43 OR 44 OR 45 OR 46 OR 47
49. 8 AND 28 AND 33 AND 48
